# Accuracy and Systematic Biases of Heart Rate Measurements by Consumer-Grade Fitness Trackers in Postoperative Patients: Prospective Clinical Trial

**DOI:** 10.2196/42359

**Published:** 2022-12-30

**Authors:** Philipp Helmer, Sebastian Hottenrott, Philipp Rodemers, Robert Leppich, Maja Helwich, Rüdiger Pryss, Peter Kranke, Patrick Meybohm, Bernd E Winkler, Michael Sammeth

**Affiliations:** 1 Department of Anaesthesiology, Intensive Care, Emergency and Pain Medicine University Hospital Würzburg Würzburg Germany; 2 Department of Software Engineering Faculty of Computer Science University of Würzburg Würzburg Germany; 3 Institute for Clinical Epidemiology and Biometry University of Würzburg Würzburg Germany; 4 Department of Applied Sciences Coburg University Coburg Germany

**Keywords:** health tracker, smartwatch, internet of things, personalized medicine, photoplethysmography, wearable, Garmin Fenix 6 Pro, Apple Watch 7, Fitbit Sense, Withings ScanWatch

## Abstract

**Background:**

Over the recent years, technological advances of wrist-worn fitness trackers heralded a new era in the continuous monitoring of vital signs. So far, these devices have primarily been used for sports.

**Objective:**

However, for using these technologies in health care, further validations of the measurement accuracy in hospitalized patients are essential but lacking to date.

**Methods:**

We conducted a prospective validation study with 201 patients after moderate to major surgery in a controlled setting to benchmark the accuracy of heart rate measurements in 4 consumer-grade fitness trackers (Apple Watch 7, Garmin Fenix 6 Pro, Withings ScanWatch, and Fitbit Sense) against the clinical gold standard (electrocardiography).

**Results:**

All devices exhibited high correlation (*r*≥0.95; *P*<.001) and concordance (*r*_c_≥0.94) coefficients, with a relative error as low as mean absolute percentage error <5% based on 1630 valid measurements. We identified confounders significantly biasing the measurement accuracy, although not at clinically relevant levels (mean absolute error<5 beats per minute).

**Conclusions:**

Consumer-grade fitness trackers appear promising in hospitalized patients for monitoring heart rate.

**Trial Registration:**

ClinicalTrials.gov NCT05418881; https://www.clinicaltrials.gov/ct2/show/NCT05418881

## Introduction

Fitness trackers are usually wrist-worn devices equipped with photoplethysmography (PPG) sensors and motion sensors, among complementary sensor units. These devices paved the way for continuous monitoring of diverse fitness parameters including various vital signs [1]. In contrast to conventional PPG measurement methods based on transmissive pulse oximetry (TPO), fitness trackers use reflective pulse oximetry. Therefore, wearing a finger clip is obsolete because both the light-emitting diode and the photodiode (light sensor) can be combined side by side in one measuring unit that can be worn, for example, on the wrist, offering more mobility to users or patients.

The option of continuous heart rate monitoring without impairing the mobility of patients opens up a range of new opportunities, especially for hospitalized patients. For example, the Early Warning Score can be calculated from heart rate and other parameters and is used for the early detection of deterioration in patients [2]. Yet, vital signs are only monitored continuously in hospitalized patients requiring intensive care, as the technical, personal, and financial requirements do not enable the current methods to be expanded to a peripheral ward. Traditional monitoring also makes patients more difficult to mobilize, which runs counter to the idea of early rehabilitation according to the guidelines of the Enhanced Recovery After Surgery. Particularly, patients undergoing surgical procedures are a vulnerable patient cohort requiring close monitoring. Recently, 2 systematic reviews demonstrated that, based on continuous measuring of vital parameters in hospitalized patients, the length of stay in the hospital [3] and, in combination with automated alerting systems, even mortality [4] could be reduced. Such reports raise the evident question to which degree fitness trackers could be used in hospitalized patients for continuous monitoring of vital signs. Due to their general availability, cost efficiency, and long battery life, fitness trackers could offer a feasible solution. To date, fitness trackers have primarily been used for sports and leisure purposes [5], but their opportunities in the continuous monitoring of various vital signs during the entire hospital stay have already been highlighted [6].

Obviously, in order to establish fitness trackers in the medical sector, a rigorous validation of their measurement accuracy is of high importance. However, so far, relatively little effort has been made in this direction, and most of the currently available trials show one or more of the following shortcomings: the study was primarily conducted with healthy volunteers [7,8], it compared different devices with each other but not with an established medical gold standard [9], it examined non–consumer-grade wearables [10], and it assessed only a very limited sample size [11].

Studies on the use of fitness trackers in a perioperative setting or among patients with multiple pre-existing diseases are rare [12] and, according to systematic reviews, also hampered by a high risk of bias [13] and suffer from low quality [14]. In particular, it has been shown that motion artifacts influence the mean absolute error (MAE) of the measurements by up to 30% [15]. In order to exclude such interferences, we evaluated the accuracy of vital signs measured by fitness trackers in resting patients. We therefore set up—for the very first time—a study that aims to benchmark the heart rate measurements of 4 consumer-grade fitness trackers against the clinical gold standard under controlled conditions in postoperative patients undergoing moderate to major surgery.

## Methods

### Study Design

The primary objective of our study is the evaluation of the accuracy of heart rate measurements by consumer-grade fitness trackers against the clinical gold standard. The study population consisted of nonsedated postoperative patients who had undergone moderate to major surgery. This prospective validation study took place at the Department of Anaesthesiology, Intensive Care, Emergency and Pain Medicine at the University Hospital Würzburg, Germany, between November 2021 and May 2022. The study protocol was designed in accordance with the guidelines for wrist-worn consumer wearables [16]. This paper presents the results of the heart rate validation in the “Monitor Trial,” registered on ClinicalTrials.org (accession No. NCT05418881).

Patients (aged ≥18 years old) scheduled for elective surgery requiring placement of an arterial line were screened prior to the procedure. Exclusion criteria for participation included critically ill patients (ie, American Society of Anesthesiologists V [ASA V]), those with a BMI of >40 kg/m^2^, outpatient surgery, infectious patients (due to hygienical regulations), those who previously participated in this study, those incapable of giving written informed consent, those who did not speak and read German, and those with extensive pathological skin lesions at the forearms or with known allergies to latex, silicone, or nickel.

### Ethical Considerations

The study protocol had been reviewed and approved by the local ethics committee of Würzburg (reference number 145/21_c). We conducted our study in accordance with good clinical practice guidelines and the Declaration of Helsinki. Our study was planned, carried out, analyzed, and interpreted independently of any industrial partners. All participants provided written informed consent before surgery took place.

### Study Procedures

Following surgical procedures, the vital parameters of study participants were continuously monitored according to hospital standards during their stay at the postanesthesia care unit (PACU). We used medical-grade TPO at the finger as well as noninvasive and invasive blood pressure monitoring and 3-lead electrocardiography (ECG), all measured by Philips devices (IntelliVue X3, Philips Healthcare). The measured parameters were streamed to a bedside patient monitor (MX750, Philips Healthcare). Simultaneously, patients were equipped with 4 different consumer-grade fitness trackers (Table 1), attached randomly to either wrist according to the manufacturer's instructions. In doing so, we aimed to eliminate any systematic bias from our results, for example, small but potentially present differences in pulse measurements between the 2 hands. During a patient’s stay at the PACU, a total of 3 on demand measurements were collected by 2 trained members of the research staff. The measured values were acquired manually from the screens of the fitness trackers and the bedside monitors (ECG and TPO) simultaneously. Patients who had no arterial line placed or those who were admitted to an intensive care unit immediately (eg, sedated, ventilated, or temporarily critically ill patients) were excluded. The placement of an arterial line ensured that only patients with moderate to major surgery were included.

In order to set up each of the fitness trackers, an anonymized, patient-unrelated user account had been created at the corresponding manufacturer. Immediately after the initial setup, the firmware of each device was updated (Table 1). Subsequently, the connection via Bluetooth and Wi-Fi was deactivated to ensure that no further firmware updates were installed during the course of the study, preventing any possible changes to algorithms from affecting the results [17,18]. Of note, although some of the manufacturers offer customized firmware for research purposes, we decided to stick to the consumer-grade firmware to enable the comparability of our results with complementary studies.

**Table 1:** Wrist-worn consumer-grade fitness trackers investigated in this trial, specified by the respective manufacturer (headquarters’ address), the device’s model, and the firmware version used for the study.

Manufacturer	Model	Firmware version
Apple	Watch 7	watchOS8.1
Fitbit	Sense	5.3 (44.128.6.12)
Garmin	Fenix 6 Pro	19.20 (0fe794a)
Withings	ScanWatch	2291

### Data Collection

Patient characteristics were recorded after performing measurements according to the guidelines for wrist-worn devices [16], including age, sex, wrist circumference, BMI, height, body weight, ASA classification, Fitzpatrick scale, and heart rhythm. As there is no generally established metrics for the density of forearm hair, we segregated the forearm hairiness of patients into 4 categories—0: no forearm hair; 1: minimal; 2: moderate; and 3: extensive hairiness. Measurements of the devices were recorded manually and transferred to an Excel (Microsoft Corp) spreadsheet later on.

### Statistical Analysis

If not further specified, all statistical analyses were carried out using standard R (version 4.2.0; R Core Team) functions and using the ggplot2 package (version 3.3.6; MIT license) for visualization. For descriptive analysis of the patient cohort, we assessed the median and the IQR of each of the attributes. In addition to the fitness tracker measurements of the heart rate, TPO as the established clinical standard for heart frequency measurement was used as a control and compared to the ECG gold standard. We assessed the measurement accuracy of each device by Bland–Altman plots [19]. After visual inspection, we excluded 5 outliers from further analysis, defined as deviations of >30 beats per minute (bpm) between the gold standard and the respective benchmarked measurement. For all of the remaining paired data points (*p_i_*,*r_i_*), the absolute error (AE) was determined as abs(*p_i_* – *r_i_*) and, inherently, the absolute percentage error as abs(*p_i _*– *r_i_*) × 100/*r_i_*, where *r_i_* corresponds to the gold standard reference measurements by ECG. Correspondingly, MAE and mean absolute percentage error (MAPE) were computed according to standard definitions using the Metrics package (version 0.1.4).

For each of the benchmarked devices, we further computed the linear regression, determined the Pearson correlation coefficient (PCC) as *r*, and used the DescTools package (version 0.99.45) to determine the Lin concordance coefficient (CCC) as *r_c_*. The PCC algorithm also provides the residual sum of squares (RSS) measure of discrepancy between the data and the prediction by the model. Comparing the distribution of benchmarked values with the distribution of gold standard reference measurements, we assessed the following hypotheses: (1) both data series are uncorrelated according to the Pearson model (standard association test, Cor-Test), (2) data are obtained from the same distribution (2-tailed Kolmogorov-Smirnov test), and (3) the 2 data vectors are shifted against each other (2-sample Mann-Whitney-Wilcoxon test). As all these tests are nonparametric, no further assumption on the nature of the compared distributions has been implied, and we generally accepted *P*<.05 as statistically significant.

## Results

### Overview of the Cohort

**Figure 1:**
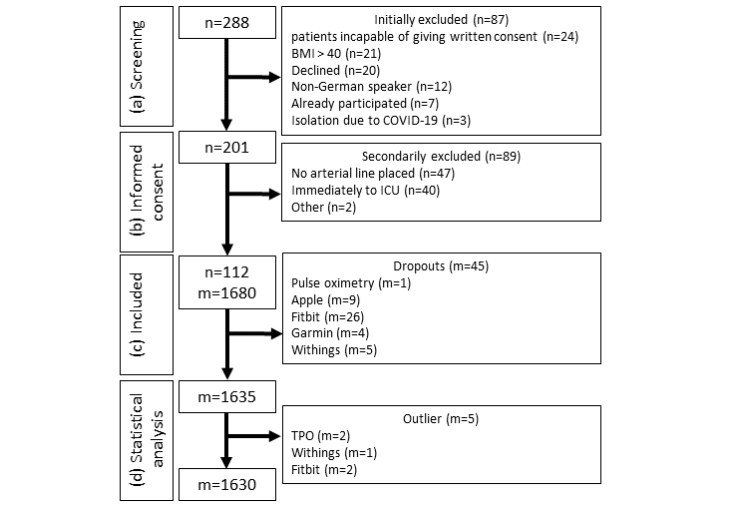
Study design. Flowchart of patient recruitment and data acquisition. After (A) screening and initially excluding patients, (B) 201 patients gave written informed consent. Of them, (C) 112 patients were successfully included in the study, resulting in 1680 benchmark measurements. Disregarding (C) missing data due to dropouts and (D) removing outliers during quality control resulted in the analyzed data set of 1630 data points. ICU: intensive care unit; m: number of measurements; n: number of patients; TPO: transmissive pulse oximetry.

During the course of the study, 288 patients were screened (Figure 1A), of whom 201 gave written informed consent (initially excluded: n=87; Figure 1B). Subsequently, a further 89 patients were excluded (Figure 1B), resulting in 112 patients successfully included in the study (Figure 1C). For each of these 112 included patients (Figure 1C), 3 attempts of measurement by each measuring method (ECG, TPO, Apple, Fitbit, Garmin, and Withings) were performed. This resulted in 2016 measurements, of which the 336 gold standard measurements (ECG) served as a reference to evaluate the remaining 1680 measurements by the benchmarked devices. Some of these measurements failed (n=45) and were classified as “dropouts.” After quality control, we removed another 5 measurements (2 TPO, 2 Fitbit, and 1 Withings), obtaining a final data set comprising 1630 data points (Figure 1D).

In our cohort, 62.5% (n=70) of participants were male and 37.5% (n=42) were female. The median age of patients was 68 years, height 172 cm, weight 77 kg, BMI 26.4 kg/m^2^, and wrist circumference 18 cm. Patients were further stratified by ASA score, skin pigmentation (Fitzpatrick scale), and a custom scale on the degree of hairiness on their forearm (Table 2, Figure S1 in Multimedia Appendix 1).

Most of the patients (n=92; 82.1%) presented with sinus heart rhythm during the measurements; hence, merely 20 (17.9%) patients presented with arrhythmias. Of them, 10 patients presented with atrial fibrillation, 5 with pacemaker-triggered ECG, 1 with bigeminus, 1 with clustered extrasystoles, 1 with a left ventricular assist device, and 2 patients were not further classified by the attending physician. No adverse or serious adverse events were observed during the study.

**Table 2:** Attributes of the patient cohort.

	Value, median (IQR)	Range (minimum-maximum)
Age (years)	68 (58-74)	24-92
Wrist circumference (cm)	18 (17-19)	15-23
BMI (kg/m^2^)	26.4 (24.05-30.18)	17.7-39.1
Height (cm)	172 (165-176)	152-192
Weight (kg)	77 (68-90)	45-122
ASA^a^	2 (2-3)	1-4
Fitzpatrick scale	2 (2-3)	1-4
Degree of forearm hair density	1 (0-2)	0-3

^a^ASA: American Society of Anesthesiologists.

### Overall Deviation

We used the 1630 valid measurements to determine the general deviation of the heart frequency measured by fitness trackers compared to the clinical gold standard. To this end, we first computed the cumulative dropout rate (CDR), taking failed measurements and data points removed during quality control into account. TPO showed the lowest dropouts (CDR<1%) among the benchmarked devices, whereas the measurements of fitness trackers yielded CDR>1%, ranging from 1.2% (Garmin) to 8.3% (Fitbit) (Table 3).

**Table 3:** Overall deviation of fitness tracker heart rate measurements and the clinical gold standard.

	Philips	Apple	Fitbit	Garmin	Withings	
Valid measurement points, n	333	327	308	332	330	
Failed measurements, n	3	9	28	4	6	
CDR^a^ (%)	0.89	2.67	8.33	1.19	1.79	
MAE^b^	0.92	1.59	2.31	2.47	1.71	
MAPE^c^ (%)	1	2	4	3	2	
Bias (95% CI)	–0.25 (–0.42 to –0.08)	0.36 (0.09 to 0.63)	0.77 (0.28 to 1.26)	–1.21 (–1.65 to –0.77)	0.05 (–0.28 to 0.40)	

^a^CDR: cumulative dropout rate.

^b^MAE: median absolute error.

^c^MAPE: mean absolute percentage error.

Next, we calculated the MAE and the relativized indicator of the MAPE between all paired measurements of a benchmarked device and the reference values. As it can be assumed that the measurements by the TPO meet clinical standards, these measurements were used as a positive control of performing the measurements accurately. As anticipated, the correlation between the measurement results of TPO and ECG was very high (*r*=0.99; *P*<.001) with an MAE of <1 bpm. TPO performs better than the fitness trackers, with an absolute deviation of ~1.5 to ~2.5 bpm on average. However, the deviation by fitness tracker measurements is overall not clinically relevant. The marginal character of the deviation is further underlined by MAPE values not reaching 5% for any of the benchmarked devices. Of note, MAPE indicators are not always proportional to the CDR indicators determined for each of the devices. Although Fitbit shows the highest CDR and MAPE, Apple exhibits the second-highest CDR but has one of the lowest MAPE (Table 3).

**Figure 2:**
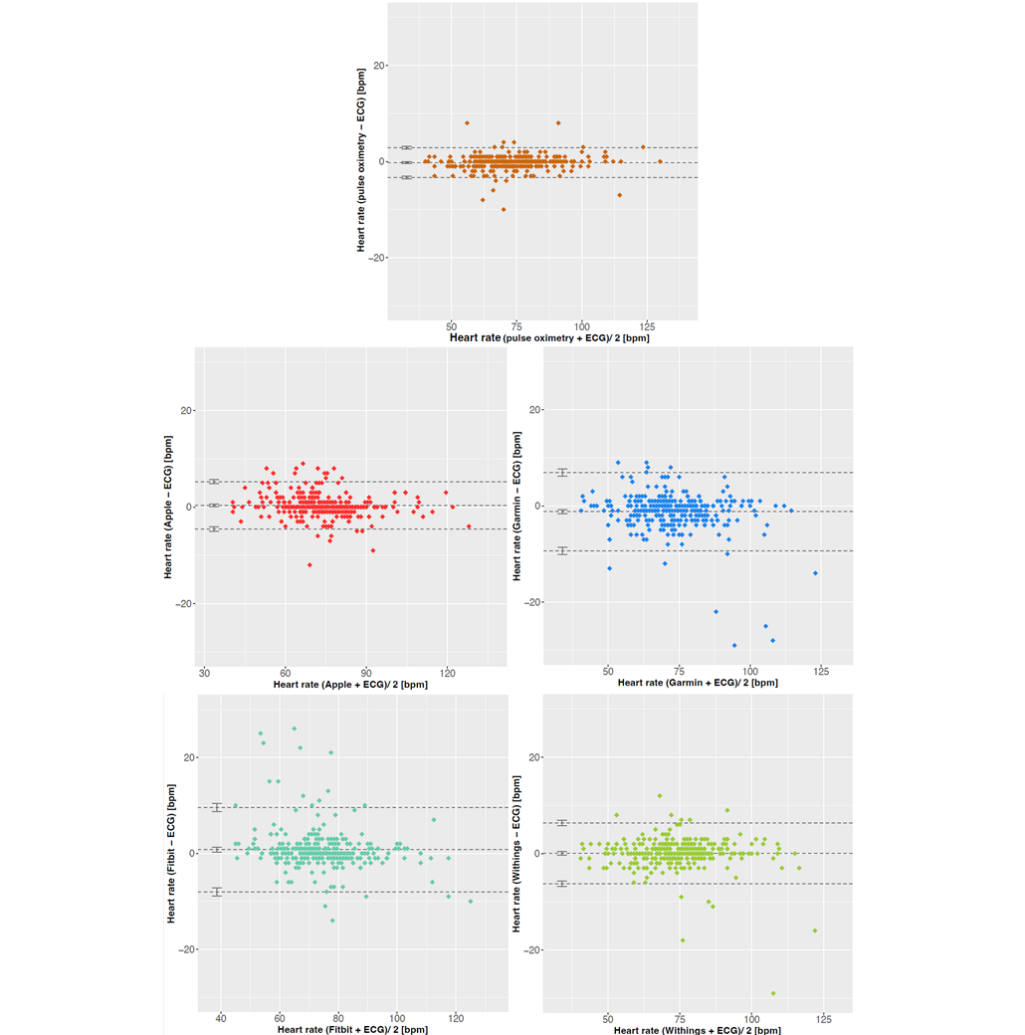
Bland–Altman plots presenting systematic bias of the investigated fitness trackers compared to ECG with the upper and lower limits of agreement and their respective CIs (upper and lower dashed line), as well as bias with the CI (middle dashed line). bpm: beats per minute; ECG: electrocardiography.

The overall bias and the SD of the measurements by the benchmarked trackers based on the ECG reference values were determined by Bland–Altman plots (Figure 2, Table S2 in Multimedia Appendix 1). The Withings tracker readings showed even less deviation from the reference than the TPO measurements (–0.25 vs 0.05; Table 3), although exhibiting an SD twice as high. Thereby, the high SD values resulted from outliers (deviation >10 bpm or even of >20 bpm), hampering particularly the Fitbit, Garmin, and Withings measurements (Figure 2). However, no systematic biases of these outliers toward high or low measurements could be identified. Overall, tracker measurements are more frequently biased to estimate higher values compared to the gold standard (ie, for Apple, Fitbit, and Withings). However, the Garmin device exhibits the absolute highest bias in the opposite direction; that is, underestimating the true heart rate. Connected by their calculation, SDs rank expectedly similar to the MAPE indicators (Table 3).

### Linear Agreement

**Figure 3:**
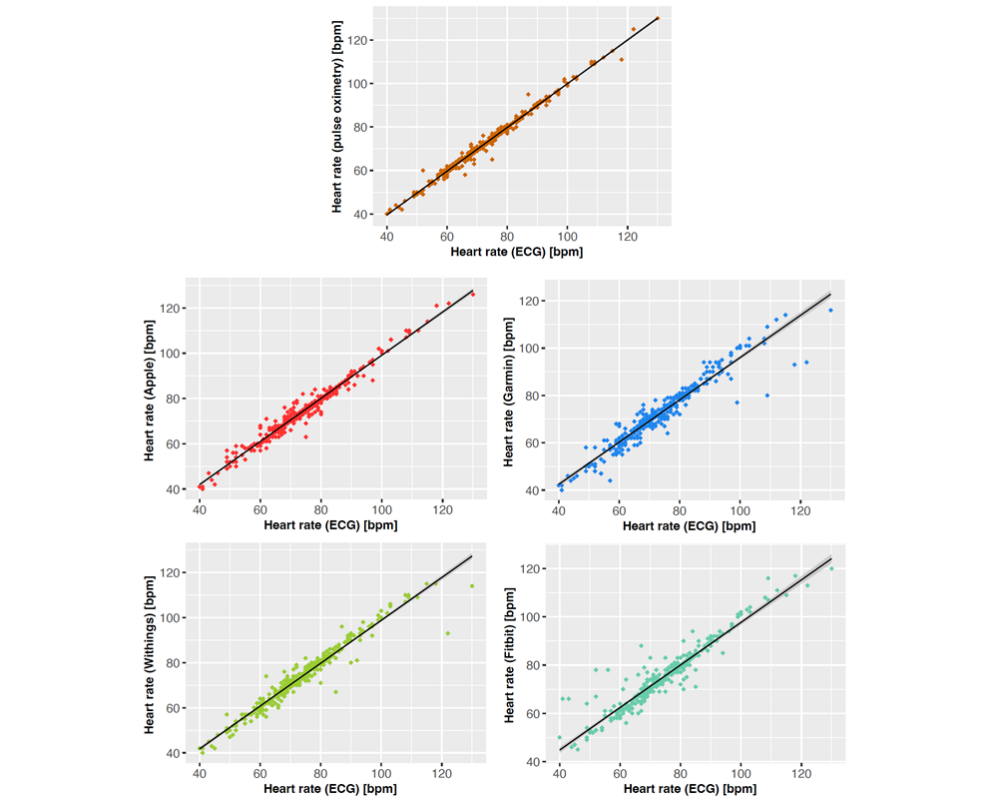
Scatter plots demonstrating good linear agreement and low dispersion between the heart rate measurements by the fitness trackers (y-axis) compared to electrocardiography (ECG) (x-axis). The respective devices are color coded. bpm: beats per minute.

In addition, the first-order correlation between benchmarked heart rate measurements and the ECG reference values was assessed. All benchmarked devices exhibited a good linear fitting of the paired data vectors, with data points scattered closely around a straight line (Figure 3). This is directly reflected by the PCCs (*r*) computed on each pair of vectors, where, in agreement with our previous results, TPO yielded the highest correlation coefficient (*r*=0.99), followed closely by Apple (*r*=0.98), Withings (*r*=0.97), Garmin (*r*=0.96), and Fitbit (*r*=0.95).

Due to the numerical proximity of the highly condensed PCC values, we also considered the RSS measures, constituting the base values for computing *r*. As can be seen from Table 4, RSS values are able to resolve more precisely the spread observed in each of the scatter plots (Figure 3), ranking the variability of measurements by the benchmarked devices more clearly from low to high: TPO (RSS=803), Apple (RSS=1830), Withings (RSS=3106), Garmin (RSS=4757), and Fitbit (RSS=5133).

The high values we observe for the PCCs indicate a strong linear fit, but do not provide further details about the slope and shift of the linear dependency. Comparing these indicators of a correspondingly regressed linear model reveals shifts of <10 and slopes of approximately 1 for each of the benchmarked devices (Table 4). We also computed CCC as a measure of deviation from direct proportionality (ie, *y*=*x*), obtaining coherent coefficients close to 1 (Table 4).

Based on these results, it is not surprising that assessing statistically the hypothesis of data being correlated (*C* test) yields a very low *P*<10^–100^ (Table 4). We used a 2-tailed Kolmogorov-Smirnov test, which supported, with a *P* value of .01, the hypothesis that the TPO measurements are pairwisely indistinguishable from the distribution of ECG reference values. This highlights a very high concordance of the measurements obtained by consumer-grade tracker devices; for example, TPO and ECG (Table 4).

**Table 4:** Assessment of linear correlation.

Indicator	TPO^a^	Apple	Fitbit	Garmin	Withings	
PCC^b^, *r* (95% CI)	0.99 (0.99-0.99)	0.98 (0.98-0.99)	0.95 (0.93-0.96)	0.96 (0.95-0.96)	0.97 (0.97-0.98)	
RSS^c^	804	1830	5133	4757	3106	
*P* value (*C* test)	5.7 × 10^–317^	2.15 × 10^–247^	2.4 × 10^–153^	1.26 × 10^–177^	2.78 × 10^–212^	
Slope	1.01	0.95	0.88	0.89	0.95	
Shift	–0.72	3.81	9.49	6.68	3.72	
CCC^d^, *r*_c_ (95% CI)	0.99 (0.99-0.99)	0.98 (0.98-0.99)	0.94 (0.93-0.95)	0.95 (0.94-0.96)	0.97 (0.97-0.98)	

^a^TPO: transmissive pulse oximetry.

^b^PCC: Pearson correlation coefficient.

^c^RSS: residual sum of squares.

^d^CCC: Lin concordance coefficient.

### Systematic Biases

We searched for systemic factors influencing the measurement accuracy of the different fitness trackers. To this end, we divided each of the attributes recorded from the patients (Table 2) into 2 subgroups (Table S1 in Multimedia Appendix 1). In theory, an adverse factor can impact the measurements of a device in two ways: (1) either the measured value is influenced negatively, resulting in a higher observed error compared to the ECG reference (ie, impact on accuracy), or (2) the device is perturbed by the factor that no measurement is produced at all (ie, impact on dropout). In order to investigate both possibilities in a comparable manner, we used, on the one hand, Mann-Whitney-Wilcoxon tests to assess the distribution of AEs in group 1 versus 2 and, on the other hand, the Fisher exact test to assess the change in dropouts between both groups.

**Figure 4:**
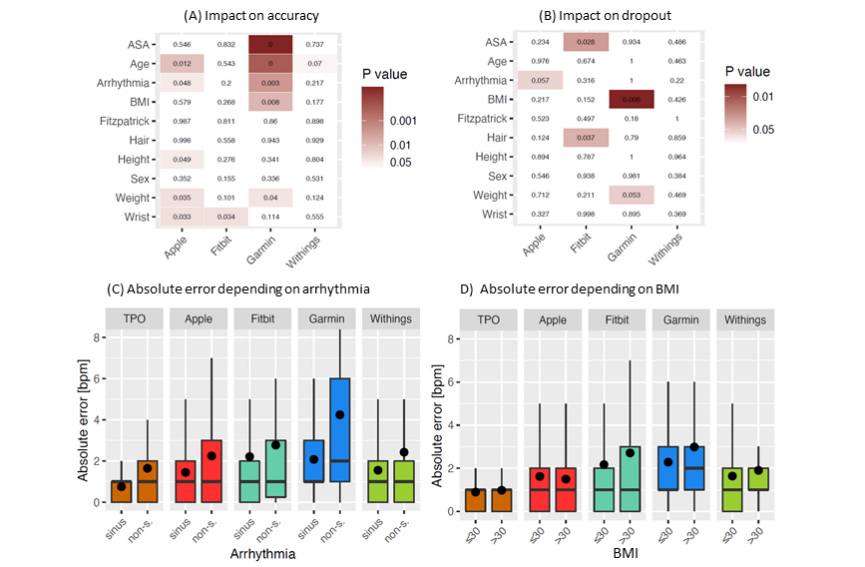
Statistical assessment of measuring failures. Upper panels: heat maps visualizing the significance level of different attributes depending on the investigated wearables (1-sided Mann-Whitney U test). The darker the color, the lower the corresponding *P* value. (A) Attributes influencing the measurement accuracies of the investigated wearables with the respective *P* values. (B) Attributes influencing the dropout rates of the investigated devices with the respective *P* values. Lower panels: Box plots for the distribution of absolute errors in binary subgroups of patients, segregated according to their health status. (C) Arrhythmia sinus versus nonsinus rhythm. (D) BMI discriminating patients with obesity from those without obesity. bpm: beats per minute; TPO: transmissive pulse oximetry.

Figures 4A and 4B summarizes the results of our analyses. Respective box plots are presented in Figure S2 in Multimedia Appendix 1. As expected, the observed deviations in accuracy as well as changes in the number of dropouts are far from statistical significance when comparing male participants with female participants. More surprisingly, dividing patients according to the Fitzpatrick scale assigned to their skin tonality did not lead to the observation of significant differences in any indicator. Using a significance threshold of *P*=.05, we identified higher ASA scores, age, arrhythmias (Figure 4C), obesity (Figure 4D), and a wrist circumference of >18 cm as confounders, significantly worsening the accuracy of some tracker measurements (Figure 4A). Concordantly, higher ASA scores, obesity, and the hair density on the forearm exhibited significant differences in the number of dropouts (Figure 4B).

The identified confounders primarily affected the Garmin tracker. Particularly, negative impacts were seen in the higher age and higher ASA cohorts (Figure 4A), and in the arrhythmia and higher BMI cohorts (Figures 4C and 4D). Further, the Apple tracker exhibits negative influences by higher age and arrhythmia, albeit of less statistical significance. However, putting these statistics on scale with the total deviation, we found the largest bias caused by cardiac arrhythmia when using the Garmin tracker corresponding to an MAE of 2.17 bpm (Figure 4C). Although the presence of some confounders also increases the MAE of Fitbit and Withings measurements (Figures 4C and 4D), these differences were in general not significantly higher than errors of measurement in the background (Figures 4A and 4B).

## Discussion

### Accuracy of the Heart Rate Measurements

The primary objective of our study was to evaluate the measurement accuracy of consumer-grade fitness trackers. According to Navalta et al [20], thresholds of MAPE≤5% and CCC≥0.90 can be considered as sufficiently high measurement accuracy. In our study, all of the benchmarked devices are within these threshold boundaries (Tables 3 and 4). In order to assess the clinical relevance of the deviations we observed between the benchmarked devices and the gold standard, we used the American National Standards Institute/Association for the Advancement of Medical Instrumentation standards for “cardiac monitors, heart rate meters, and alarms” (Association for the Advancement of Medical Instrumentation 2002) based on which an AE<5 bpm or relative (ie, percentage) error of <10% is required [21]. Our results (Figure 2) demonstrate that for each of the benchmarked devices, >92% of the measurements are within these limits (98.5% of TPO, 97.6% of Apple, 92.9% of Fitbit, 94% of Garmin, and 96.7% of Withings measurements). For upcoming trials, standardization of these thresholds is highly desirable in order to objectively decide on an “acceptable measurement accuracy” of a PPG-based device.

Overall, the measurement accuracy of consumer-grade fitness trackers is marginally inferior to that of TPO readings in postoperative patients while being at rest. However, the consumer-grade devices exhibit a wider dispersion in their measurements (Figure 3), as well as higher dropout rates than TPO (Table 3). Since the measurement accuracy of fitness trackers from different manufacturers depends on various technical details, we empirically tested potential confounders of heart rate measurements. Although we identified some factors that significantly decreased the accuracy of measurement (Figure 4), the observed deviations did not reach a clinically relevant level (MAE<5 bpm). To summarize, our observations support the use of fitness trackers for heart rate monitoring in postsurgical immobilized patients.

In general, the comparability of our results with previous studies is hampered by differences in methodological approaches, study designs, differences of the investigated collectives, etc. A systematic review estimated an overall MAPE between 1% and 7% for heart rate measurements of the Apple Watch [22]. In healthy test participants, Lauterbach et al [23] demonstrated an acceptable heart rate measurement accuracy with a bias <–1 bpm for the Garmin Fenix 5x plus. In patients with pre-existing cardiovascular disease, the Apple Watch Sport showed an MAE of 6.34 bpm compared to a 12-lead ECG, leading Falter et al [24] to conclude clinically acceptable accuracy. Focusing on the use of the Apple Watch 6 in patients with lung diseases or cardiovascular diseases, heart rate measurements showed a bias of –0.11 bpm and achieved a PCC of *r*=0.98 compared to standard finger pulse oximeters [25]. A further study comparing Apple Watch against pulse oximeters, including 100 pulmonary prediseased patients in a sitting position, demonstrated a concordance of *r_c_*=0.995 in heart rate measurements [26]. Additionally, when comparing the Apple Watch against a telemetry monitor (CARESCAPE Monitor, GE Healthcare) in patients with atrial fibrillation and obstructive sleep apnea, authors concluded acceptable measurement accuracy [27]. On the other hand, wrist-worn devices were considered unsuitable for supraventricular tachycardia detection, if these last for less than 60 seconds [28]. Additionally, the Fitbit tracker, when compared to the clinical gold standard in patients requiring intensive care, exhibited a bias of –4.7 bpm (95% CI –4.91 to –4.44) and a relatively low correlation of *r*=0.74 [29]. To our best knowledge, there are currently no comparable results from other studies investigating heart rate measurement accuracy based on PPG signals by the Apple Watch 7, the Garmin Fenix 6 Pro, the Withings ScanWatch, and the Fitbit Sense. To date, there is equally poor evidence on the clinical use of further parameters measured by fitness trackers, for example, heart rate variability, blood pressure, oxygen saturation, and cardiac output.

### Wearables in Digital Health Care

As part of clinical trials, an increasing number of systems that enable continuous monitoring of patients’ vital signs are finding their way into clinical settings. In particular, wearables were used for early diagnostics in clinical studies during the COVID-19 pandemic [30], demonstrating that an infection can be detected by wearables even before a positive nose swab [31]. Techniques to detect certain cardiac arrhythmias with consumer-grade devices are currently being validated [32]. A randomized trial involving older adult patients in this area of application demonstrated that the detection rate of atrial fibrillation is increased by one order of magnitude compared to the standard care group [33]. Moreover, ongoing efforts on developing artificial intelligence models are using data collected from consumer-grade wearables in order to detect and to predict cardiovascular-related diseases [34]. A further meta-analysis focusing on the early detection of sepsis concluded that even mortality is reduced (risk ratio 0.56) by automated alerts when comparing artificial intelligence–based continuous vital sign monitoring systems to standard care [4]. However, wearables provide the possibility of early diagnosis and therefore of initiating timely therapies, but obviously do not alone constitute a therapeutic tool [35]. Furthermore, the compliance of patients using such wearables is of fundamental importance. In this regard, an average wear time of 23.1 hours per day has been reported in patients with dementia, who also demonstrated a high degree of satisfaction according to a survey [36]. Other challenges that need to be resolved in order to implement wearable systems at a large scale concern the financing concepts. Although the devices are significantly more cost efficient than the current standard monitoring, concrete concepts will require further development.

### Limitations

There are several limitations to our study. First, even though some cardiac applications of the devices we used are approved by the US Food and Drug Administration, manufacturers generally discourage using them for diagnostic testing in a medical setting. Next, some important technical details—particularly the length of the time interval over which the heart rate is measured by the consumer-grade trackers as well as the delay between measuring and displaying the result—are not disclosed publicly by the manufacturers. This could result in hidden biases when time matching the measurements of different fitness trackers with each other and with the gold standard reference values. With respect to this concern, we also could not fully address the question of up to which degree dropouts in the measurements of fitness trackers are related to technical problems, problems in the usage, or internal quality control mechanisms of the underlying algorithms.

We collected 3 consecutive measurements per patient during a comparatively short interval. Therefore, conclusions about long-term use are clearly beyond the scope of this trial. Furthermore, our study is underpowered to assess the measurement accuracy of the devices at extreme values of the heart rate because 78.2% of our ECG data can be considered of regular heart rate (60-90 bpm), 11.9% are bradycardic (<60 bpm), and 9.9% are tachycardic (>90 bpm). Similarly, although our results support the hypothesis of higher BMI values impairing the measurement performance, our data ultimately cannot elucidate the effects of obesity to its full impact because our study design did not include patients with a BMI of >40 kg/m^2^. Additionally the median of the skin pigmentation in our cohort corresponds to Fitzpatrick scale 2, therefore, no final conclusions can be drawn about the impact of dark skin on the accuracy of the trackers. Since we focused on resting patients in the supine position, no conclusions can be drawn about the measurement accuracy of mobile patients [8]. Therefore, future studies are essential to evaluate wearables in mobile patients.

### Conclusions

We summarize that consumer-grade wearables demonstrate promising accuracy for heart rate monitoring in postsurgical patients after moderate to major surgery. The confounders identified in this study did not affect heart rate measurements to a clinically relevant extent.

## Appendix

Multimedia Appendix 1 Supplementary figures and tables.

## Abbreviations

AE: absolute error
ASA: American Society of Anesthesiologists
bpm: beats per minute
CCC: Lin concordance coefficient
CDR: cumulative dropout rate
ECG: electrocardiography
MAE: mean absolute error
MAPE: mean absolute percentage error
PACU: postanesthesia care unit
PCC: Pearson correlation coefficient
PPG: photoplethysmography
RSS: residual sum of squares
TPO: transmissive pulse oximetry

## Data Availability

Deidentified data can be requested for noncommercial research to qualified academic institutions. A formal request for data access must be sent to the principal investigator (helmer_p@ukw.de), including a standard data use agreement and guarantee that data will be used for research purposes only.
