# Supplementary material for: Accuracy and Systematic Biases of Heart Rate Measurements by Consumer-Grade Fitness Trackers in Postoperative Patients: Prospective Clinical Trial
Source: J Med Internet Res. 2022 Dec 30;24(12):e42359. doi: 10.2196/42359 (PMC9840097; doi:10.2196/42359)
Supplement: Multimedia Appendix 1 [file jmir_v24i12e42359_app1.docx]

# **Supplementary Information**

Accuracy and Systematic Biases of Heart Rate Measurements by Consumer-Grade Fitness Trackers in Postoperative Patients: a prospective clinical trial

Philipp Helmer, Sebastian Hottenrott, Philipp Rodemers, Robert Leppich, Maja Helwich, Rüdiger Pryss, Peter Kranke, Patrick Meybohm, Bernd Winkler, Michael Sammeth

##

## **Supplementary Figures**

**Figure S1.** Bar charts of the collected characteristics of the population. The x-axis shows the corresponding absolute values of the different parameters and the y-axis the relative distribution.

| 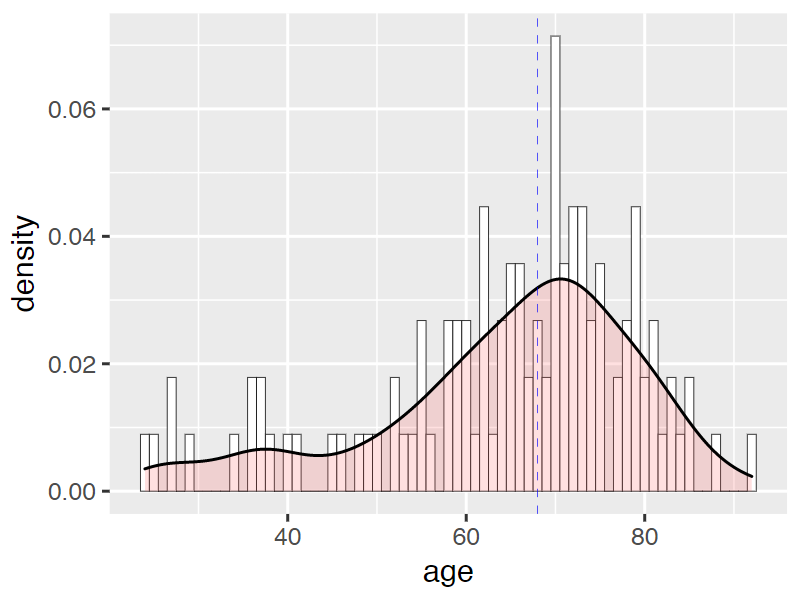 | 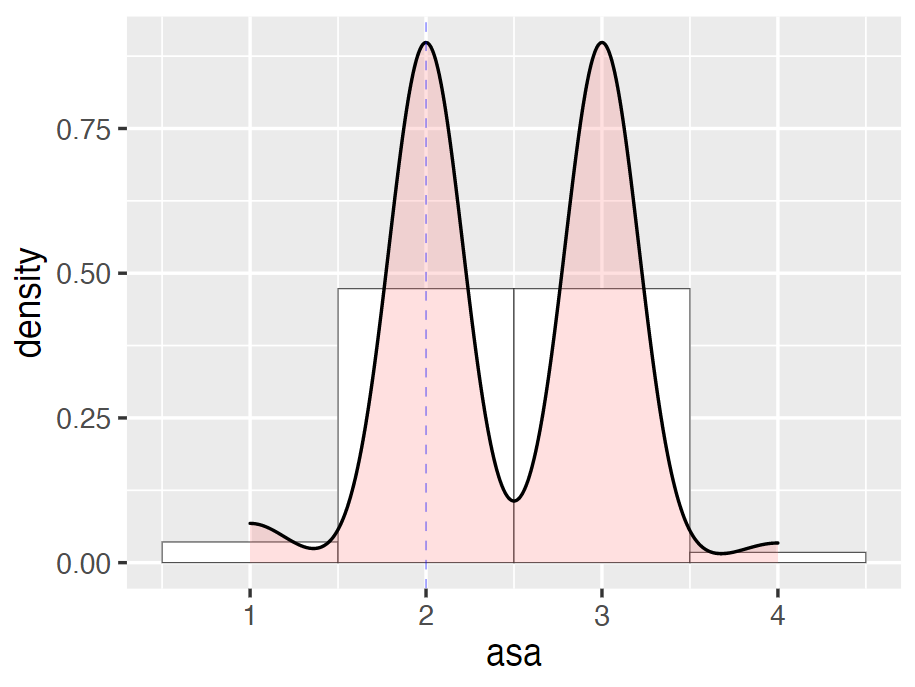 |
| --- | --- |
| 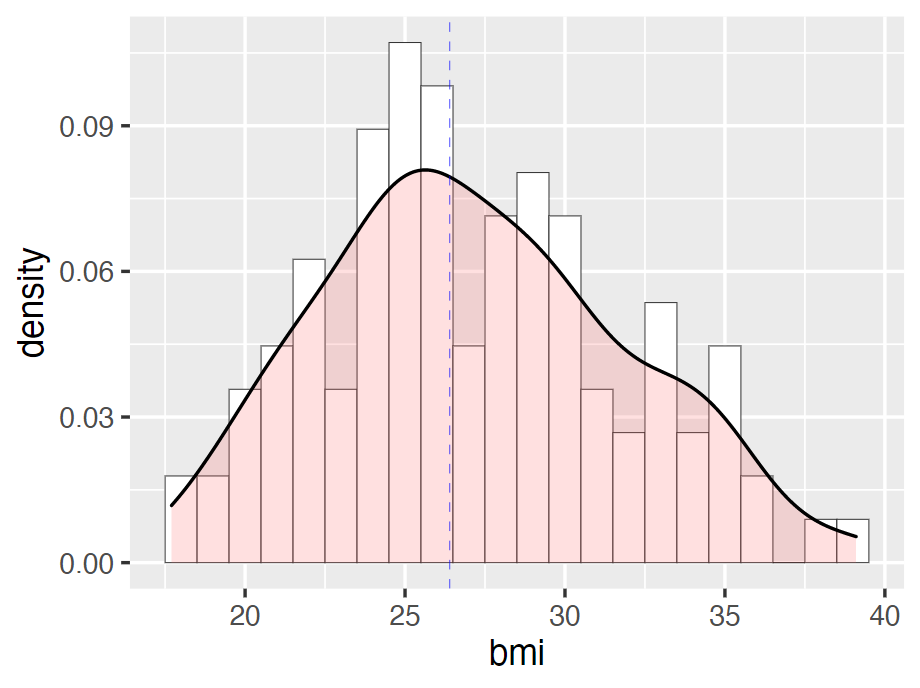 | 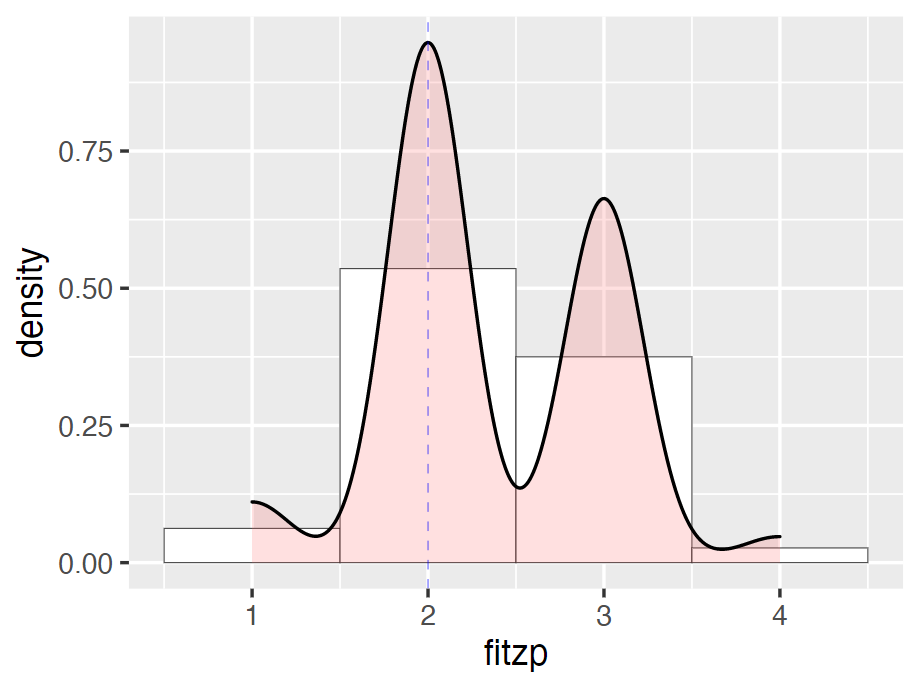 |
| 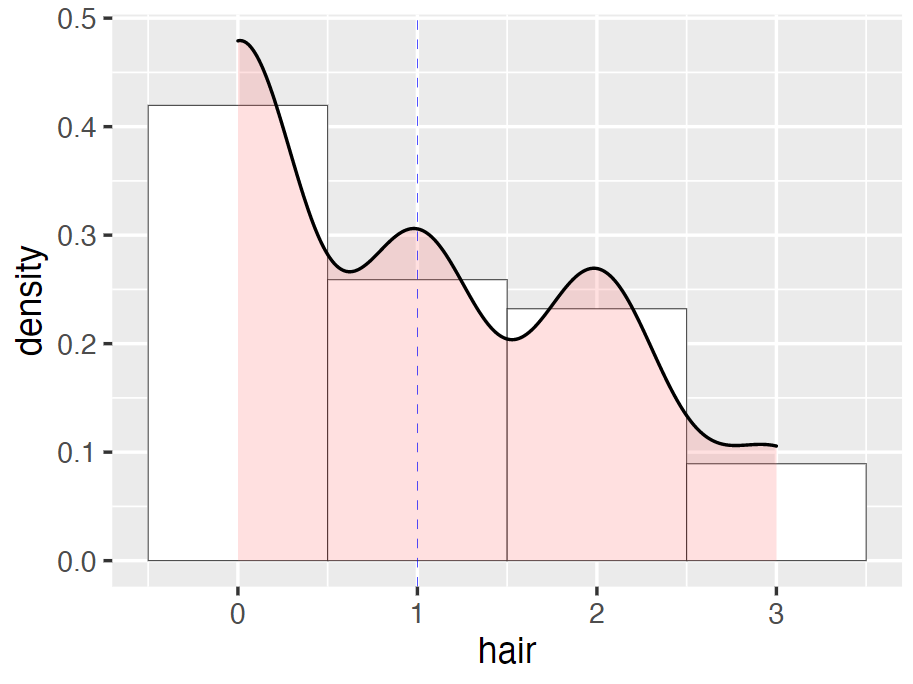 | 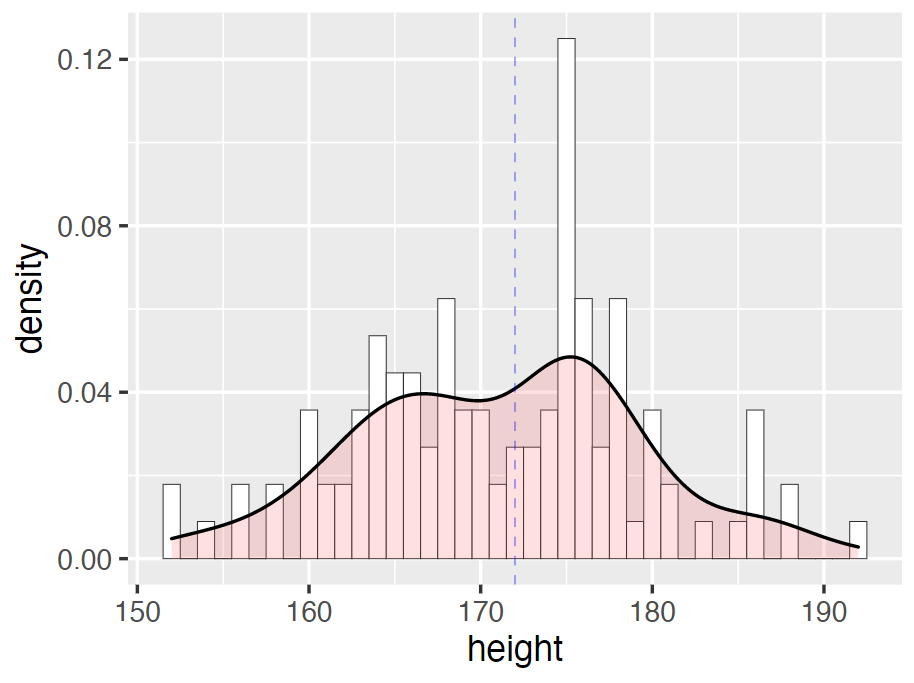 |
| 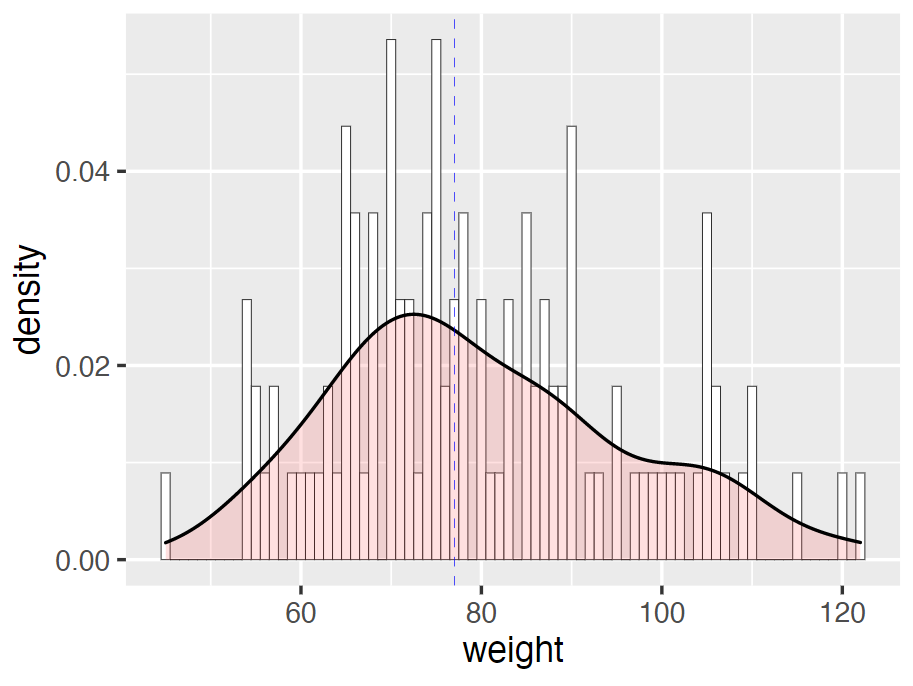 | 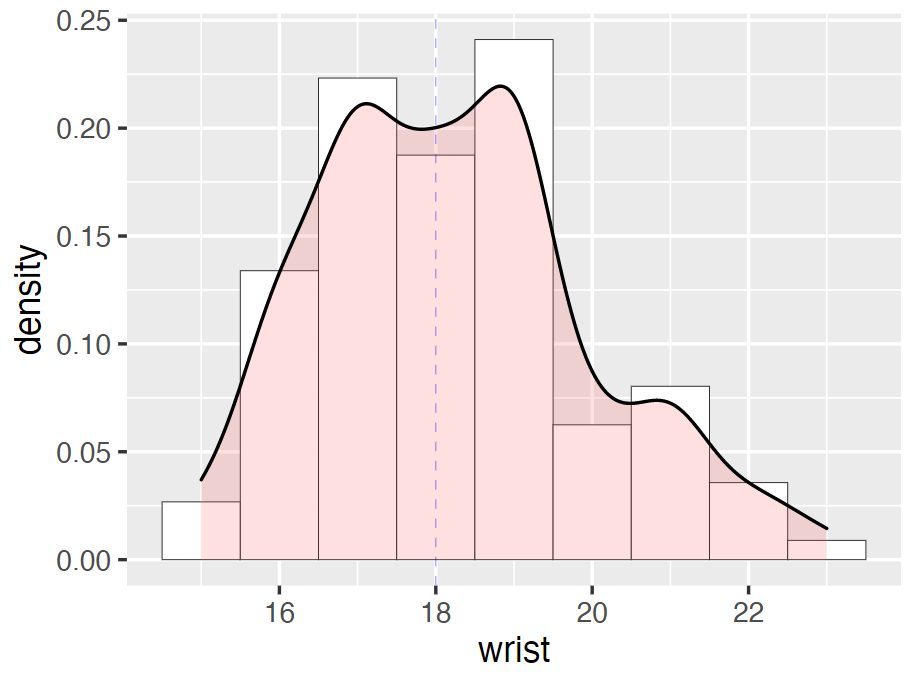 |
|  | |

**Figure S2.** The absolute errors of the investigated attributes are shown divided into the respective subgroups and plotted using boxplots. The respective devices are colour coded. The median is shown as a dash in the boxplots, whereas the mean is shown as a dot.

| 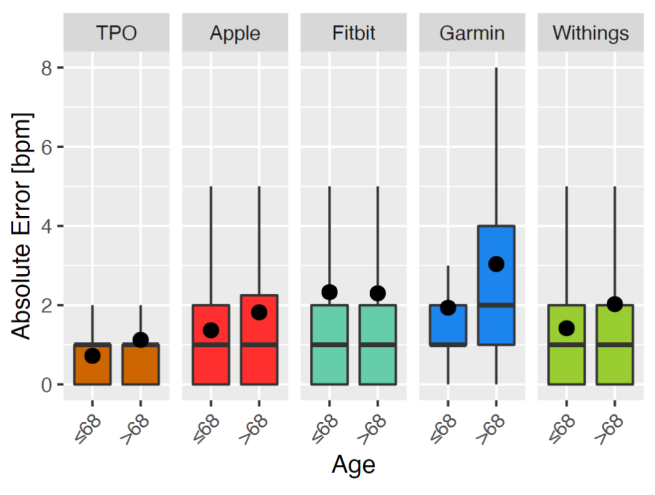 | 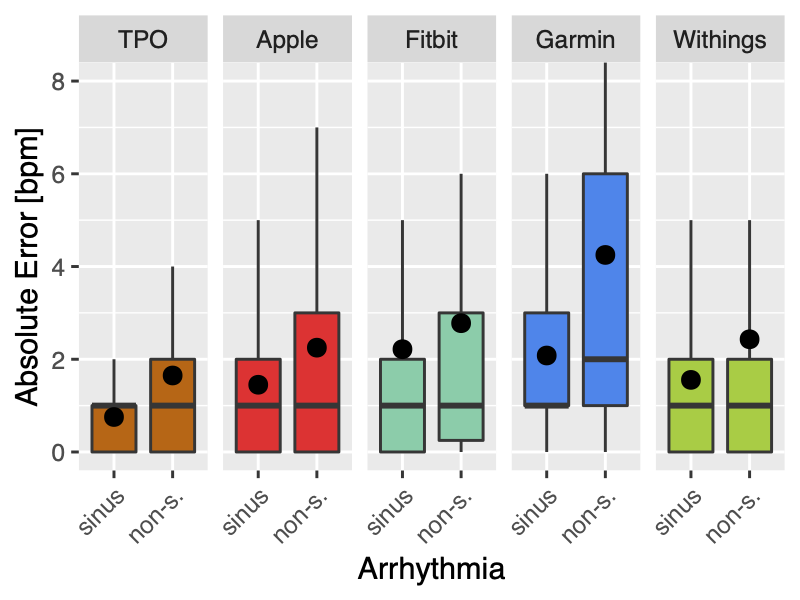 |
| --- | --- |
| 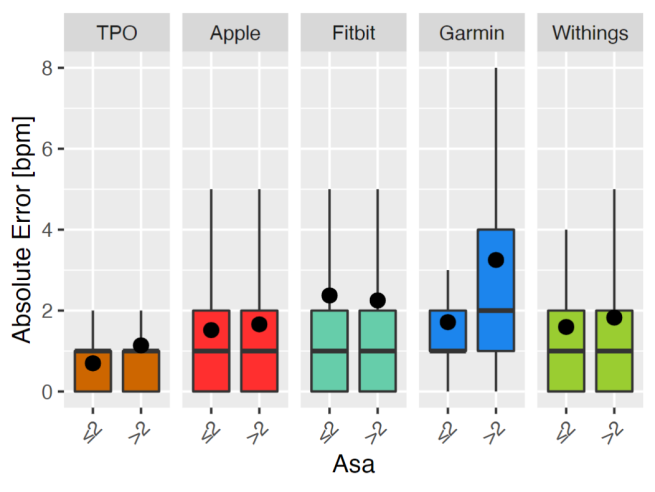 | 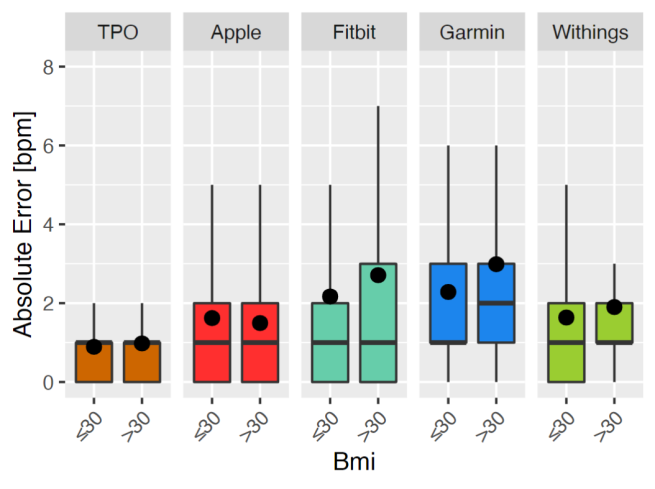 |
| 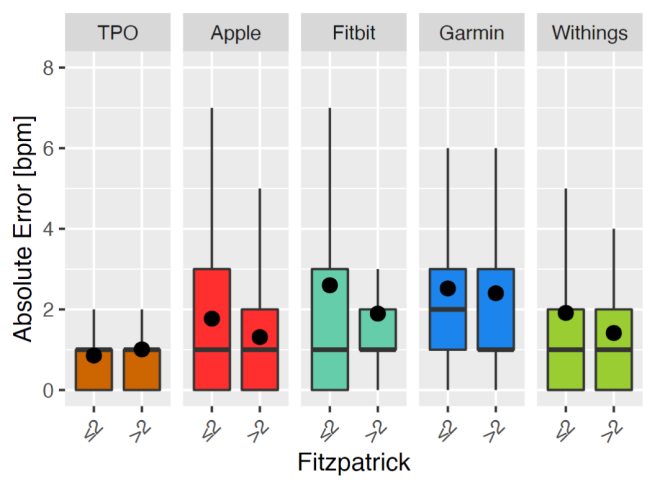 | 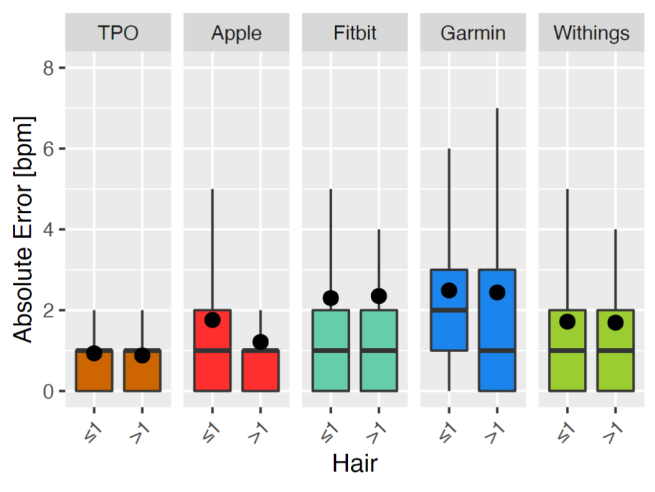 |
| 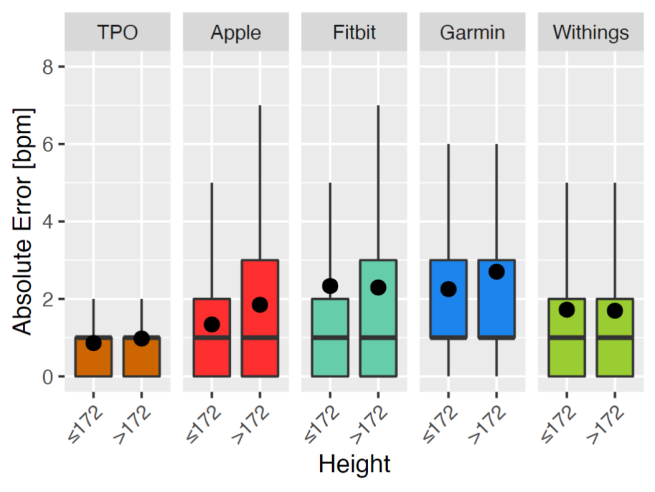 | 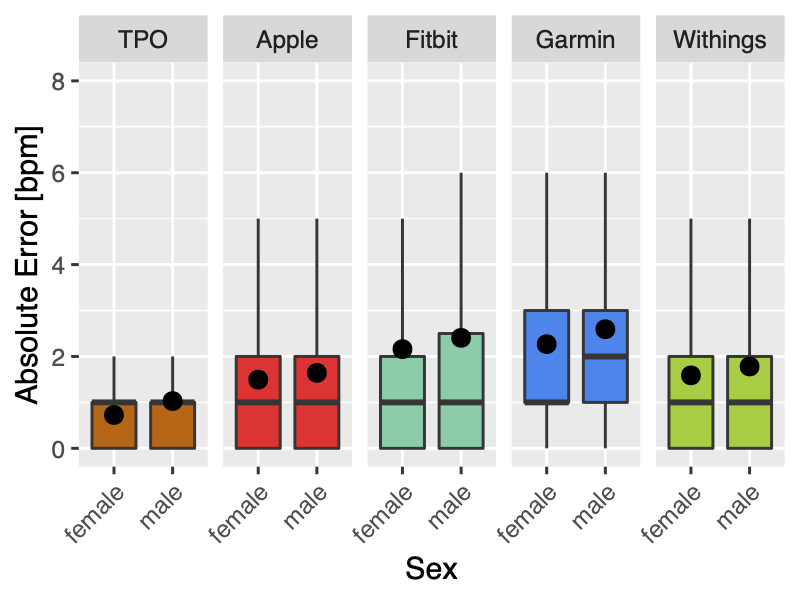 |
| 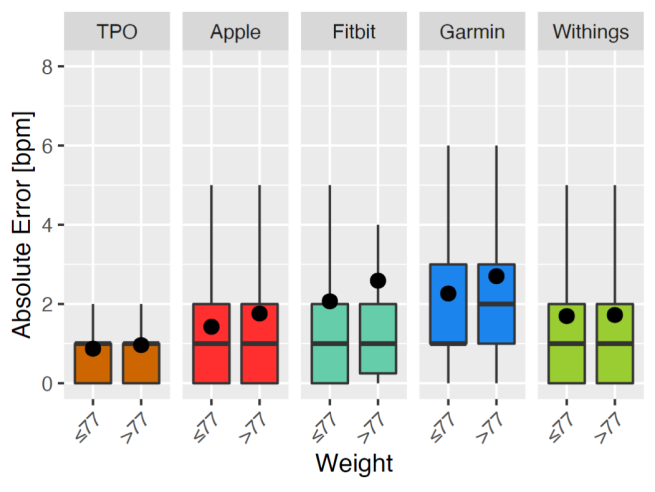 | 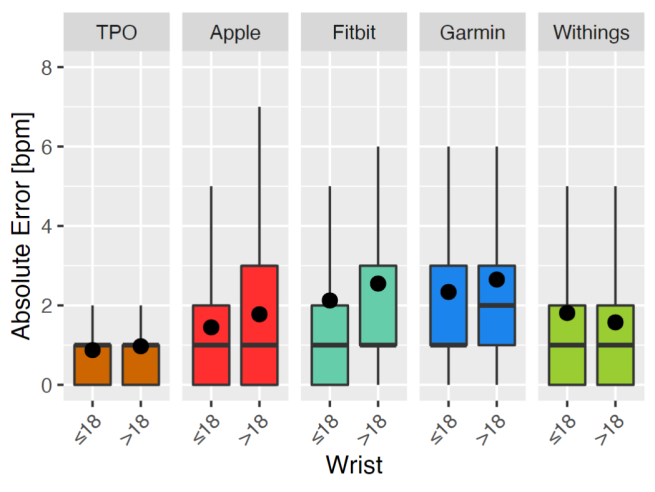 |
|  | |

## **Supplementary Tables**

**Table S1.** Attributes among the analysed subgroups with corresponding group size. Overall sample size n=112.

| **attributes** | **Group 1** | **Group 2** |
| --- | --- | --- |
| Age [years] | ≤68 (n=58) | >68 (n=54) |
| Arrhythmia | Sinus rhythm (n=92) | Non Sinus rhythm (n=20) |
| ASA | ≤2 (n=57) | >2 (n=55) |
| BMI [kg/m2] | ≤30 (n=81) | >30 (n=31) |
| Fitzpatrick-Scale | ≤2 (n=67) | >2 (n=45) |
| Hair density | ≤1 (n=76) | >1 (n=36) |
| Hight [cm] | ≤172 (n=58) | >172 (n=54) |
| Sex | Female (n=42) | Male (n=70) |
| Weight [kg] | ≤77 (n=58) | >77 (n=54) |
| Wrist cirumferences [cm] | ≤18 (n=64) | >18 (n=48) |

#### **Table S2.** Numerical results of the Bland-Altman plots for the different devices. Systematic bias with confidence interval, standard deviation, as well as the lower and upper limits of agreement with corresponding confidence intervals are given.

| **Manufactures** | **Philips** | **Apple** | **FitBit** | **Garmin** | **Withings** |
| --- | --- | --- | --- | --- | --- |
| **Bias with CI** | -0.25  [-0.42; -0.08] | 0.36  [0.09; 0.63] | 0.77 [0.28; 1.26] | -1.21 [-1.65; -0.77] | 0.05 [-0.28; 0.40] |
| **SD** | 1.56 | 2.46 | 4.4 | 3.86 | 3.15 |
| **Lower limit of agreement (CI)** | -3.37 [-3.66; -3.08] | -4.56 [-5.02; -4.09] | -8.03 [-8.88; -7.17] | -9.35 [-10.11; -8.59] | -6.24 [-6.83; -5.65] |
| **Upper limit of agreement (CI)** | 2.87 [2.58; 3.16] | 5.27 [4.81; 5.73] | 9.56 [8.71; 10.42] | 6.92 [6.16; 7.68] | 6.35 [5.76; 6.94] |

SD=standard deviation. CI=confidence interval.
